# Iatrogenic obstructive acute kidney injury due to suprapubic catheterization

**DOI:** 10.1093/omcr/omac011

**Published:** 2022-02-19

**Authors:** Lucas Jacobs, Ayemane Salif, Victor Calderon Plazarte, Ibrahim Alcan, Maxime Taghavi

**Affiliations:** Nephrology Clinic, Internal Medicine Department, Brugmann University Hospital, Brussels, Belgium; Nephrology Clinic, Internal Medicine Department, Brugmann University Hospital, Brussels, Belgium; Urology Department, Brugmann University Hospital, Brussels, Belgium; Radiology Department, Brugmann University Hospital, Brussels, Belgium; Nephrology Clinic, Internal Medicine Department, Brugmann University Hospital, Brussels, Belgium

## MANUSCRIPT

**Figure 1:**
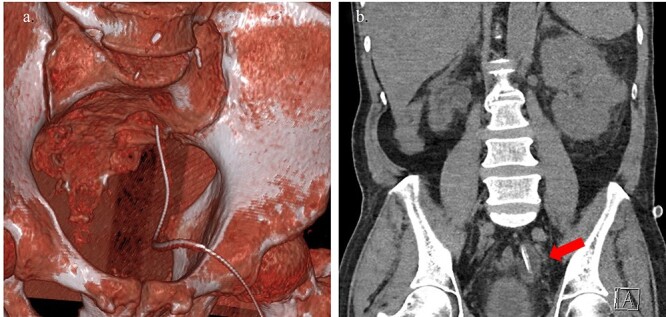
(a) 3D-reconstruction CT image shows the suprapubic catheter; (b) frontal CT image shows the right atrophic kidney, as well as the insertion of the suprapubic catheter from the bladder to the left ureter with distal hydroureter and the inflated balloon (red arrow).

We describe the case of a 66-year-old man with stage G3b chronic kidney disease (CKD). CKD was secondary to recurrent stricture of the bulbar urethra, urinary outlet obstructions, recurrent urinary tract infection (UTI) and atrophic right kidney. Iterative replacements of the suprapubic cystostomy (SPC) were performed. Six hours after last SPC replacement, the patient presented to the emergency room with impaired general status, chills and 39°C pyrexia. He was tachycardic (120 beats/min) without hypotension or need for oxygen. The work-up revealed severe acute kidney injury (AKI) and UTI. Biochemistry showed elevated creatinine and urea (13.7 and 294 mg/dl, respectively), hyperkalemia (6.6 mmol/l) and metabolic acidosis (bicarbonate 10 mmol/l). Arterial blood gas analysis showed normal pH and lactate levels (lactate 3.5 mmol/l; reference range < 2 mmol/l). Urine and blood cultures were positive for *Escherichia coli*. Computed tomography (CT) of the abdomen showed severe left hydronephrosis and hydroureter due to the obstruction of the left distal ureter by the distal extremity of the SPC and the inflated balloon (Figure 1). Evolution was favorable after SPC’s repositioning, after that the intravenous perfusion was set to match his urine output and a 7-day course of antibiotics. His daily urine volumes decreased from 4300 to 2400 cc at discharge.

SPC is a common procedure for bladder drainage. Acute ureteral obstruction is a rare complication related to the procedure [1]. SPC can cause recurrent ureteral obstruction and urosepsis [2]. To avoid the occurrence of obstructive AKI, clinician should evaluate SPC’s theoretical length needed [3]. Also, prognosis of AKI depends on its stage, but also on its duration [4], and this case highlights the importance of rapid imaging in case of AKI for early detection and treatment of obstruction.

## CONSTENT

Informed consent was obtained from the patient.

## References

[1] Elmoheen A, Saqr M, Salem W, Bashir K, Hagras A. Suprapubic catheter migration: a review of a rare complication. Case Rep Urol 2021;2021:8816213. 33489411 10.1155/2021/8816213 PMC7803400

[2] Adeyemo B, Makovitch S, Foo D. A peculiar complication of suprapubic catheterization: recurrent ureteral obstruction and hydronephrosis. J Spinal Cord Med 2013;36:166–9. 23809534 10.1179/2045772312Y.0000000080 PMC3595967

[3] Chautemps N, Milesi C, Forgues D, Adra A-L, Morin D, Cambonie G. Anuric acute renal failure after suprapubic catheterization. Arch Pediatr 2012;19:422–4. 22365502 10.1016/j.arcped.2012.01.010

[4] Kellum JA, Romagnani P, Ashuntantang G, Ronco C, Zarbock A, Anders H-J. Acute kidney injury. Nat Rev Dis Primers 2021;7:52. 34267223 10.1038/s41572-021-00284-z

